# The IL-10 homologue encoded by cyprinid herpesvirus 3 is essential neither for viral replication in vitro nor for virulence in vivo

**DOI:** 10.1186/1297-9716-44-53

**Published:** 2013-07-16

**Authors:** Ping Ouyang, Krzysztof Rakus, Maxime Boutier, Anca Reschner, Baptiste Leroy, Maygane Ronsmans, Guillaume Fournier, Sophie Scohy, Bérénice Costes, Ruddy Wattiez, Alain Vanderplasschen

**Affiliations:** 1 Immunology-Vaccinology (B43b), Department of Infectious and Parasitic Diseases (B43b), Faculty of Veterinary Medicine, University of Liège, Liège, B–4000, Belgium; 2 Proteomic and Microbiology, CISMa, University of Mons, Place du parc, 20, Mons B-7000, Belgium; 3 Delphi Genetics SA, rue Antoine de Saint-Exupéry, 5, Gosselies B-6041, Belgium; 4 Femalon S.A, Rue du Travail, 16, Grâce-Hollogne B-4460, Belgium

## Abstract

Cyprinid herpesvirus 3 (CyHV-3), a member of the family *Alloherpesviridae*, is the causative agent of a lethal disease in common and koi carp. CyHV-3 ORF134 encodes an interleukin-10 (IL-10) homologue. The present study was devoted to this ORF. Transcriptomic analyses revealed that ORF134 is expressed as a spliced gene belonging to the early-late class. Proteomic analyses of CyHV-3 infected cell supernatant demonstrated that the ORF134 expression product is one of the most abundant proteins of the CyHV-3 secretome. To investigate the role of ORF134 in viral replication in vitro and in virulence in vivo, a deleted strain and a derived revertant strain were produced using BAC cloning technologies. The recombinant ORF134 deleted strain replicated in vitro comparably to the parental and the revertant strains. Infection of fish by immersion in water containing the virus induced comparable CyHV-3 disease for the three virus genotypes tested (wild type, deleted and revertant). Quantification of viral DNA by real time TaqMan PCR (in the gills and the kidney) and analysis of carp cytokine expression (in the spleen) by RT-qPCR at different times post-infection did not revealed any significant difference between the groups of fish infected with the three virus genotypes. Similarly, histological examination of the gills and the kidney of infected fish revealed no significant differences between fish infected with ORF134 deleted virus versus fish infected with the control parental or revertant strains. All together, the results of the present study demonstrate that the IL-10 homologue encoded by CyHV-3 is essential neither for viral replication in vitro nor for virulence in common carp.

## Introduction

Koi herpesvirus (KHV), also known as cyprinid herpesvirus 3 (CyHV-3; species *Cyprinid herpesvirus 3*, genus *Cyprinivirus*, family *Alloherpesviridae*, order *Herpesvirales*), is the etiological agent of an emerging and mortal disease in common (*Cyprinus carpio carpio*) and koi (*Cyprinus carpio koi*) carp [1,2]. Since its emergence, in the late 1990s, this highly contagious and dreadful disease has caused severe economic losses in both common and koi carp culture industries worldwide [3-5].

The genome of CyHV-3 comprises a linear double-stranded DNA sequence of ~295 kbp [6], similar to that of cyprinid herpesvirus 1 and 2 (CyHV-1 and CyHV-2) [7,8], but larger than those of other members of the order *Herpesvirales* which generally range from 125 to 240 kbp. Phylogenetic analysis of the CyHV-3 genome sequence led to its classification in the new family *Alloherpesviridae* encompassing herpesviruses of fish and amphibians [9,10]. The CyHV-3 genome contains 155 potential protein-coding open reading frames (ORFs), some of which have relatives in other herpesviruses, and a few of which have relatives in poxviruses, iridoviruses and other large DNA viruses [6,8,11]. Interestingly, CyHV-3 genome encodes proteins potentially involved in immune evasion mechanisms such as, for example, TNF receptor homologues (encoded by ORF4 and ORF12) and an IL-10 homologue (encoded by ORF134) [6].

Cellular IL-10 has been described in a wide range of vertebrate species, including fish [12,13]. It is a pleiotropic immunomodulatory cytokine with both immunostimulating and immunosuppressive properties [14]; however, IL-10 is generally described as an immunosuppressive cytokine. It inhibits expression of a large number of cytokines as, for example, TNF-α, IFN-γ, IL-1β, IL-2, IL-3, IL-6, and MHC class II [15-17]. Many viruses exploit the immunosuppressive properties of IL-10 to evade immune recognition either by up-regulation of host IL-10 or by expression of virally encoded IL-10 homologues (vIL-10s) [14,18,19].

Virally encoded IL-10 homologues have been reported in members of the *Poxviridae* family and the *Herpesvirales* order [19-21]. Among the *Herpesvirales* order, vIL-10s have been described in members of the *Herpesviridae* (e.g. human cytomegalovirus [HCMV] and Epstein-Barr virus [EBV]) and more recently in the family *Alloherpesviridae* (Anguilid herpesvirus 1 [AngHV-1] and CyHV-3) [22]. While the role of vIL-10s has been demonstrated in the pathogenesis of one *Poxviridae* and one *Herpesviridae*[23-25]; this has not yet been investigated in the family *Alloherpesviridae*. However, a very recent study suggested that the IL-10 homologue encoded by CyHV-3 ORF134 could play a role in the pathogenesis. Firstly, it has been demonstrated that this ORF is transcribed in infected fish maintained at permissive and even restrictive temperature [26]. Secondly, it has been shown that injection of CyHV-3 ORF134 mRNA into zebrafish embryos increased the number of lysozyme-positive cells to a similar degree as zebrafish IL-10 [26]; an effect that was inhibited by down regulation of the IL-10 receptor long chain using a specific morpholino [26].

The present study was devoted to CyHV-3 ORF134 encoding an IL-10 homologue. In vitro studies demonstrated that ORF134 is expressed as a spliced early-late gene and that its expression product is the second most abundant viral protein in the CyHV-3 secretome. Taking advantage of the recent BAC cloning of CyHV-3 as an infectious bacterial artificial chromosome (BAC), a strain deleted for ORF134 and a derived revertant strain were produced. Comparison of these strains demonstrated that ORF134 is essential neither for CyHV-3 replication in vitro nor for virulence in common carp.

## Materials and methods

### Cells and viruses

*Cyprinus carpio* brain cells (CCB) were cultured in minimum essential medium (MEM) (Invitrogen, Merelbeke, Belgium) containing 4.5 g/L glucose (D-glucose monohydrate; Merck, Darmstadt, Germany) and 10% fetal calf serum (FCS). Cells were cultured at 25 °C in a humid atmosphere containing 5% CO_2_. The CyHV-3 FL strain was isolated from the kidney of a fish that died from CyHV-3 infection (CER, Marloie, Belgium) [27].

### Determination of ORF134 kinetic class of transcription

These experiments were performed as described elsewhere [28]. Briefly, monolayers of CCB cells in 24-well plates were pre-incubated for 2 h before infection with cycloheximide (CHX) (100 μg/mL) (Sigma-Aldrich, Saint Louis, Missouri, USA) or phosphonoacetic acid (PAA) (300 μg/mL) (Sigma-Aldrich), the inhibitors of de novo protein synthesis or viral DNA polymerase, respectively. After removal of the medium, cells were infected with CyHV-3 FL strain at a multiplicity of infection (MOI) of 0.1 plaque forming unit (PFU) per cell in presence of inhibitors. After an incubation of 2 h, cells were overlaid with fresh medium containing the inhibitors. At 6, 8 and 12 h after inoculation cells were harvested and treated for RT-PCR analysis of gene expression (see below). CyHV-3 ORF3 (immediate early [IE]), ORF55 (early [E]) and ORF78 (late [L]) were used as reference gene of the three kinetic classes [29,30].

### Transcriptional analysis by RT-PCR

Cytoplasmic RNA was isolated from cells using the RNeasy Mini Kit (Qiagen, Venlo, Netherlands) with on column DNase I digestion. cDNA was synthetized from 1 μg of RNA using iScript™ cDNA Synthesis Kit (Bio-Rad, Nazareth Eke, Belgium). Finally, PCRs were performed with the primers listed in Table 1 (see RT-PCR column).

**Table 1:** Primers and probes.

**Targeted gene**	**Primer/probe name**			**Sequence (5’- 3’)**	**Accession n°/ reference**
**Primers for PCR and RT-PCR**		PCR	RT-PCR		
CyHV-3 ORF3	ORF3InF		•	TATGCCCACATGATGCTGTT	DQ657948
	ORF3InR		•	CAGTCAGACCCTTCCTCTGC	
CyHV-3 ORF55	ORF55InF	•		AGCGCTACACCGAAGAGTCC	
	ORF55stopR	•		TCACAGGATAGATATGTTACAAG	
	ORF55ATGF		•	ATGGCTATGCTGGAACTGG	
	ORF55InR		•	GGCGCACCCAGTAGATTATG	
CyHV-3 ORF78	ORF78InF		•	TGGACGACGAACACCCTTC	
	ORF78InR		•	GGTAGAGGGTACAACCACG	
CyHV-3 ORF132	ORF132InF		•	GGATCCGTTTTCTGGGTCTG	
	ORF132InR		•	CTCAATCCCTCACCGACCTC	
CyHV-3 ORF133	ORF133InF		•	GACGAGATCCCTATCCGCAG	
	ORF133InR		•	GACCTCGGGTATGGTCGGTA	
CyHV-3 ORF134	ORF134stopF		•	TCAATGTTTGCGCTTGGTTTTC	
	ORF134ATGR		•	ATGTTCCTTGCAGTGCTAC	
	ORF134InF	•		GGTTTCTCTTTGTAGTTTTCCG	
	ORF134InR	•		CACCCCAACTTTTGAGACAAC	
	ORF134outseqF	•		GTCAACATGGACGAGCGTGA	
	ORF134outseqR	•		GTGGGGATATCAAACACGCA	
CyHV-3 ORF135	ORF135InF		•	ACACCACCAACGAGACATGC	
	ORF135InR		•	CTTTTCGGACCAGAAGACCG	
Carp β-actin	Actin-F		•	ATGTACGTTGCCATCCAGGC	M24113
	Actin-R		•	GCACCTGAACCTCTCATTGC	
**Primers for amplification of recombination cassettes**					
H1-*gal*K-H2 cassette	134 *gal*K F			ATGTTCCTTGCAGTGCTACTAACCG	Warming et al. [34]
				CGACCATCTTCTTCGAGGCTCGGGG	
				CCTGTTGACAATTAATCATCGGCA	
	134 *gal*K R			TCAATGTTTGCGCTTGGTTTTCATG	
				TTCTTGACGTCTTTTGCGACCAGGA	
				TCAGCACTGTCCTGCTCCTT	
H1-ORF134-H2	H1F			GCTCATCAATCGCAGCAGCA	DQ657948
cassette					
	H2R			CAAGCCATTATCCTGTTGGG	
**Primers and probes for real-time TaqMan PCR quantification of CyHV-3 genome**					
CyHV-3 ORF89	KHV-86F			GACGCCGGAGACCTTGTG	AF411803
	KHV-163R			CGGGTTCTTATTTTTGTCCTTGTT	
	KHV-109P			(6FAM)CTTCCTCTGCTCGGCGAGCACG(BHQ1)	
Carp glucokinase	CgGluc-162F			ACTGCGAGTGGAGACACATGAT	AF053332
	CgGluc-230R			TCAGGTGTGGAGCGGACAT	
	CgGluc-185P			(6FAM)AAGCCAGTGTCAAAATGCTGCCCACT(BHQ1)	
**Primers for RT-qPCR analysis of carp gene expression**					
40S	40S-F			CCGTGGGTGACATCGTTACA	AB012087
	40S-R			TCAGGACATTGAACCTCACTGTCT	
IL-1β	IL-1β-F			AAGGAGGCCAGTGGCTCTGT	AJ245635
	IL-1β-R			CCTGAAGAAGAGGAGGCTGTCA	
TNF-α1 and 2	TNF-α1 and 2-F			GCTGTCTGCTTCACGCTCAA	AJ311800
	TNF-α1 and 2-R			CCTTGGAAGTGACATTTGCTTTT	
CXCa	CXCa-F			CTGGGATTCCTGACCATTGGT	AJ421443
	CXCa-R			GTTGGCTCTCTGTTTCAATGCA	
IL-10	IL-10-F			CGCCAGCATAAAGAACTCGT	AB110780
	IL-10-R			TGCCAAATACTGCTCGATGT	
IFNγ-2	IFNγ-2-F			TCTTGAGGAACCTGAGCAGAA	AM168523
	IFNγ-2-R			TGTGCAAGTCTTTCCTTTGTAG	
IL-6	IL-6M17-F			CACATTGCTGTGAGGGTGAA	AY102633
	IL-6M17-R			GCATCCATAGGCTTTCTGCT	

Underlined: 50bp corresponding to CyHV-3 sequence.

### Production of concentrated cell supernatant

CCB cells were infected with CyHV-3 FL strain at a MOI of 0.05 PFU per cell using serum free culture medium. Cell supernatants were collected 72 h post-inoculation and then submitted to two cycles of centrifugation at 4 °C (clarification at 2000 *g* for 15 min followed by pelleting of viral particles at 100 000 *g* for 2 h through a 30% sucrose gradient). The supernatant was then concentrated 25-fold by centrifugation (2000 *g*, 75 min, 4 °C) through an Amicon Ultra-15 centrifugal filter unit (3K NMWL; Merck Millipore, Billerica Massachusetts, USA) and stored at −80 °C until use.

### 2D-LC MS/MS proteomic approach

Proteomic analyses were performed using 2D-LC MS/MS workflow as described previously [31]. Briefly, proteins were reduced at 4 °C for 1 h with 10 mM DTT and alkylated by incubation with 25 mMiodoacetamide at 4 °C for 1 h in the dark. Proteins were recovered through acetone precipitation and digested with trypsin at an enzyme:substrate ratio of 1:50 in 50 mM NH_4_HCO_3_ overnight at 37 °C. Tryptic peptides (25 μg) were analysed by bidimensional (SCX-RP) chromatography and online MS/MS, as described elsewhere [32], except that only 3 successive salt plugs of 25, 100 and 800 mM NH_4_Cl were used. Peptides were analyzed using the “peptide scan” option of an HCT ultra ion Trap (Bruker, Evere, Belgium), consisting of a full-scan mass spectrometry (MS) and MS/MS scan spectrum acquisitions in ultrascan mode (26 000 m/z sec-1). Peptide fragment mass spectra were acquired in data-dependent AutoMS (2) mode with a scan range of 100–2,800 m/z, three averages, and 5 precursor ions selected from the MS scan 300–1500 m/z. Precursors were actively excluded within a 0.5 min window, and all singly charged ions were excluded. Peptide peaks were detected and deconvoluted automatically using Data Analysis 2.4 software (Bruker). Mass lists in the form of Mascot Generic Files were created automatically and used as the input for Mascot MS/MS Ions searches of the NCBInr database release 20120809 using an in-house Mascot 2.2 server (Matrix Science). The default search parameters used were: Taxonomy = Bony vertebrates or Cyprinivirus; Enzyme = Trypsin; Maximum missed cleavages = 1; Fixed modifications = Carbamidomethyl (C); Variable modifications = Oxidation (M); Peptide tolerance ± 1.2 Dalton (Da); MS/MS tolerance ± 0.6 Da; Peptide charge = 2+ and 3+; Instrument = ESI-TRAP. All data were also searched against the NCBI bony vertebrate database in order to detect host proteins. Only proteins identified with p value lower than 0.05 were considered, and single peptide identifications were systematically evaluated manually. In addition, the emPAI [33] was calculated to estimate protein relative abundance in the culture supernatant.

### Production of CyHV-3 ORF134 recombinants

**Figure 1:**
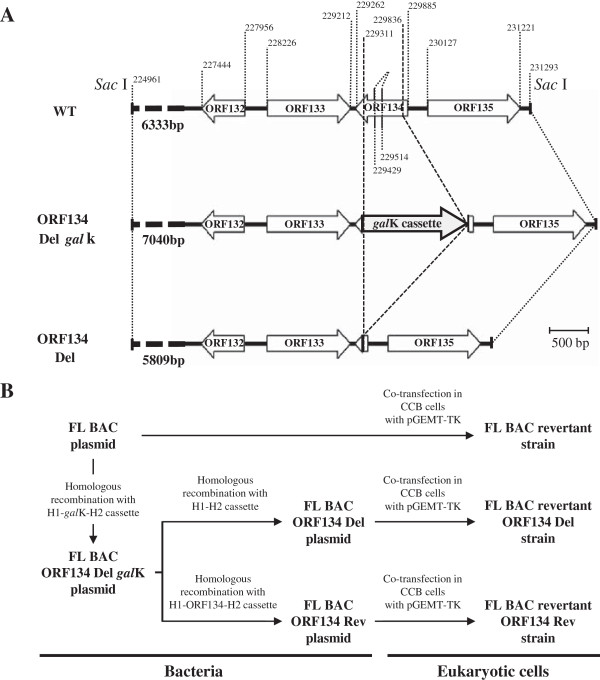
**Schematic representation of the strategy used to produce CyHV-3 FL BAC ORF134 recombinants.** (**A**) The region of CyHV-3 genome encoding ORF134 is illustrated for wild type (WT), ORF134 Del *gal*K and ORF134 Del genotypes. (**B**) Flow chart of stages performed to produce FL BAC ORF134 recombinant plasmids and to reconstitute virus strains.

CyHV-3 recombinants were produced using prokaryotic recombination technologies (Figure 1). The FL BAC plasmid was used as parental plasmid [27]. In this plasmid, the BAC cassette is inserted in ORF55 encoding thymidine kinase (TK). ORF134 recombinant plasmids were produced using two-steps galactokinase gene (*gal*K) positive/negative selection in bacteria as described previously [34]. The first recombination process (*gal*K positive selection) consisted to replace ORF134 by *gal*K resulting in the FL BAC ORF134 Del *gal*K plasmid. Recombination was achieved using the H1-*gal*K-H2 recombination cassette (Figure 1b) which consisted of the *gal*K gene flanked by 50-bp sequences homologous to CyHV-3 genome regions flanking ORF134 deletion (Figure 1a). H1-*gal*K-H2 recombination cassette was produced by PCR (primers 134 *gal*K F and 134 *gal*K R) using the p*gal*K vector as template. Primer 134 *gal*K F consisted of nucleotides 229836–229885 (50bp) of CyHV-3 genome and 1–24 (24bp) of the p*gal*K vector. Primer 134 *gal*K R consisted of nucleotides 229262–229311 (50bp) of the CyHV-3 genome and nucleotides 1212–1231 (20bp) of the pgalK vector (Table 1). The 50-bp sequences of the H1-*gal*K-H2 corresponding to CyHV-3 genome were used to target homologous recombination in bacteria. The second recombination process (*gal*K negative selection) consisted to remove the *gal*K gene (FL BAC ORF134 Del plasmid) or to replace the *gal*K gene by CyHV-3 wild type ORF134 sequence (FL BAC ORF134 Rev plasmid) (Figure 1). The FL BAC ORF134 Del plasmid was obtained by recombination with the H1-H2 cassette (Figure 1b). This cassette was synthesized and consisted of 200 bp of CyHV-3 genome upstream and downstream of ORF134 deletion, respectively. The FL BAC ORF134 Rev plasmid was produced by recombination with the H1-ORF134-H2 cassette. This cassette was produced by PCR (primers H1F and H2R) using CyHV-3 FL DNA as template corresponding to nucleotides 229057–229076 and nucleotides 230056–230075 of CyHV-3 genome, respectively. To reconstitute infectious virus encoding a wild type TK locus (removal of the BAC cassette), the BAC plasmids (FL BAC, FL BAC ORF134 Del and FL BAC ORF134 Rev) were co-transfected with the pGEMT-TK plasmid (molecular ratio, 1:75) into CCB cells [27]. Plaque negative for enhanced green fluorescent protein (EGFP) expression (the BAC cassette encodes an EGFP expression cassette) were picked and amplified.

### Southern blotting

Southern blot analysis of recombinant viruses was performed as described previously [27,35]. PCRs were performed to produce ORF55 probe (primers ORF55InF and ORF55stopR) and ORF134Del probe (primers ORF134InF and ORF134InR) using the CyHV-3 FL genome as a template (Table 1).

### Multi-step growth curves

Triplicate cultures of CCB cells were infected at a MOI of 0.5 PFU per cell. After an incubation period of 2 h, cells were washed with phosphate-buffered saline (PBS) and then overlaid with Dulbecco’s modified essential medium (DMEM, Invitrogen) containing 4.5 g of glucose/liter and 10% FCS. Supernatant of infected cultures was harvested at successive intervals after infection and stored at −80 °C. The amount of infectious virus was determined by plaque assay on CCB cells as described previously [35].

### Fish

Common carp (*Cyprinus carpio carpio*) (CEFRA, University of Liège, Belgium), were kept in 60-liter tanks at 24 °C. Microbiological, parasitical and clinical examinations of the fish just before the experiments demonstrated that these fish were fully healthy.

### CyHV-3 inoculation of carp

For viral inoculation mimicking natural infection, fish were kept for 2 h in water containing CyHV-3. At the end of the incubation period, fish were returned to larger tanks. In some experiments, fish that survived the primary infection were challenged 42 days after inoculation by cohabitation with fish that were infected by immersion in water containing 200 PFU/mL of the FL strain just before their release into the tank to be challenged. Two freshly infected fish were released per tank to be challenged. The animal study was accredited by the local ethics committee of the University of Liège, Belgium (Laboratory accreditation N°1610008, protocol N°810).

### Quantification of virus genome copies in organs by real-time TaqMan PCR

Virus genome quantitation was performed by real-time TaqMan PCR as described elsewhere [36]. The primers and the probes used are presented in Table 1. Two sets of primers were used to amplify fragments of CyHV-3 ORF89 and carp glucokinase genes. The amplicons were cloned into the pGEM-T Easy vector and the resulting plasmids were used to generate standard curves by running reactions with 10^1^ to 10^10^ plasmid molecules. DNA was isolated using a DNA mini kit (Qiagen) from 25 mg of organs stored at −80 °C in RNAlater® (Invitrogen). The reaction mix contained 1 × iQSupermix (Bio-Rad), 200 nM of each primer, 400 nM of fluorescent probe and 250 ng of DNA. The analyses were performed using a C1000 Touch Thermal cycler (Bio-Rad). All real-time TaqMan PCRs for CyHV-3 DNA were run with equal amounts of DNA estimated by the real-time TaqMan PCR performed on carp glucokinase gene.

### Quantification of carp gene expression in spleen by RT-qPCR

Total RNA was isolated from spleens stored at −80 °C in RNALater® (Ambion®, Invitrogen, Merelbeke, Belgium) using TRI reagent® (Ambion®, Invitrogen), including DNase I digestion and RNA purification using RNeasyMinElute Cleanup Kit (Qiagen). cDNA was synthetized from 1 μg of RNA using iScriptcDNA Synthesis Kit (Bio-Rad). The primers used for RT-qPCR were described previously [37] and are listed in Table 1. The RT-qPCR master-mix was prepared as follows: 1 × IQ™ SYBR® Green Supermix (Bio-Rad), 200 nM of each primer, 5 μL of 25 × diluted cDNA and sterile water to a final volume of 25 μL. The amplification program included an initial denaturation at 95 °C for 10 min, followed by 40 cycles with denaturation at 95 °C for 15 s, annealing at 58 °C for 30 s and elongation at 72 °C for 30 s. At the end, the dissociation stage was performed (95 °C for for 10 s) and the melt curve was obtained by increasing the temperature from 60 °C to 95 °C with a rate of 0.5 °C per 5 s. Fluorescence data from RT-qPCR experiments were analyzed using the CFX96 real-time system and exported to Microsoft Excel. The threshold cycle (Ct) was determined using the Auto method for all runs. The expression of analyzed genes was calculated using the 2^-ΔΔCt^ method [38]. The 40S ribosomal protein S11 was used as a reference gene.

### Histological analysis

Organs from mock-infected or infected carp were fixed in 4% buffered formalin and embedded in paraffin blocks. Sections of 5 μm were stained with haematoxylin and eosin prior to microscopic analysis [39].

### Statistical analyses

Multi-step growth curves data expressed as mean titer ± standard deviation (SD) were analyzed for significance of differences (*p*< 0.05) using one-way ANOVA. The differences in mortality induced by the CyHV-3 strains tested were analyzed using Kaplan and Meier survival analysis. Significant differences (*p*< 0.05) in virus load between fish infected with the different CyHV-3 strains at each sampling point were assessed using one-way ANOVA followed by Holm-Sidak test when data were normally distributed, or with the non-parametric Kruskal–Wallis test followed by Tukey test when they were not. Significant differences (*p*< 0.05) in RT-qPCR gene expression between CyHV-3 infected and mock-infected fish, as well as between fish infected with different CyHV-3 strains at each sampling point were assessed using one-way ANOVA followed by Holm-Sidak test in cases where the data were normally distributed, or with the non-parametric Kruskal–Wallis test followed by Dunn’s test when they were not

## Results

### CyHV-3 ORF134 kinetic class of expression

**Figure 2:**
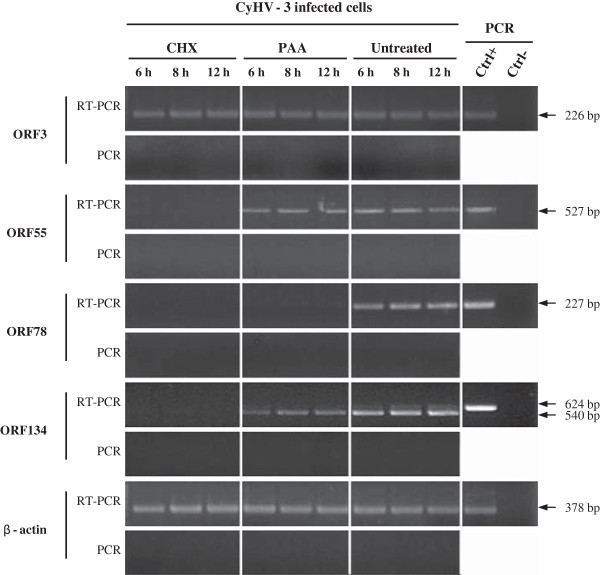
**Determination of ORF134 kinetic class of transcription.** CCB cells were infected with CyHV-3 FL strain, in absence (Untreated) or presence of CHX or PAA as described in Materials and methods. At the indicated time post-infection, expression of IE ORF3, E ORF55, L ORF78, ORF134 and carp β-actin was studied by a RT-PCR approach. On the left RT-PCR or PCR represent PCR products generated when the RT was performed or omitted from the reactions, respectively. On the right, control PCR reactions were performed using genomic DNA as template (Ctrl+) or no template (Ctrl-).

Two independent studies have demonstrated that CyHV-3 ORF134 is transcribed during viral replication in vitro thereby meeting the criteria for being a gene [26,29]. It has been predicted to contain an 84 bp intron flanked by 2 exons encoding a 179 amino acid product (GenBank accession number DQ657948). Here, we used CHX and PAA to identify the transcriptional class of ORF134 (Figure 2). This experiment revealed that ORF134 expression is prevented by CHX and reduced but not prevented by PAA treatments, suggesting that ORF134 is an E-L gene. ORF3, ORF55 and ORF78 were used as controls in this experiment; the results presented in Figure 2 confirmed that they are IE, E and L genes, respectively. The absence of contaminant viral DNA in the mRNA preparations was confirmed by the absence of a PCR product when the reverse transcriptase was omitted from the reactions. Furthermore, the estimated molecular size of the major ORF134 RT-PCR product revealed that it was derived, from the amplification of cDNA (540 bp) rather than from the viral genome (624 bp). This observation is consistent with the earlier description of the ORF134 as a spliced gene [40,41]. However, a minor product corresponding to the unspliced transcript of ORF134 was also observed (see the faint 624 bp band in Figure 2). The classification of ORF134 as an E-L gene is consistent with the results published recently by Ilouze et al. who concluded that ORF134 is an E gene [29]. It is also consistent with the E expression reported for other vIL-10s [40,41].

### CyHV-3 secretome

**Figure 3:**
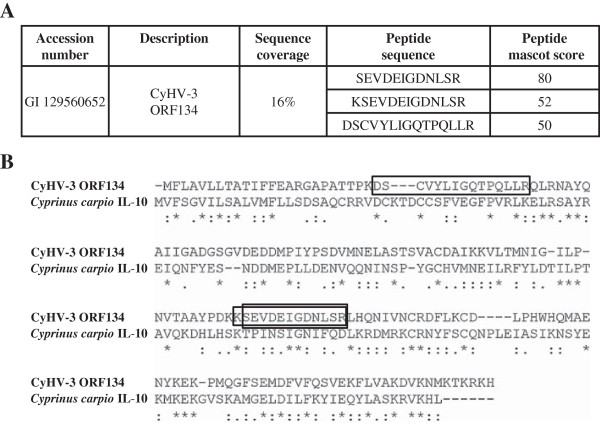
**Identification of CyHV-3 ORF134 by 2D-LC MS/MS in the supernatant of CyHV-3 infected CCB cells.** (**A**) Data collected on ORF134 through 2D-LC MS/MS analysis of cell culture supernatant. (**B**) Sequential alignment of CyHV-3 ORF134 and *Cyprinus carpio* IL-10. Sequence coverage: detected peptides are presented in rectangles.

While two independent studies have previously shown that ORF134 is transcribed during viral replication [26,29], it is still to be determined whether ORF134 encodes a protein secreted from infected cells. To address this question, concentrated supernatant was produced from CyHV-3 infected CCB cultures and analyzed by 2D-LC MS/MS. Viral and cellular proteins identified by this approach are listed in Table 2. This list was restricted to proteins identified with p value lower than 0.05 as determined by the MASCOT program. Five viral and 46 cellular proteins were detected. CyHV-3 ORF12 and ORF134 were amongst the most abundant proteins in the sample as revealed by their relatively high emPAI scores (1.49 and 1.02, respectively). Only two cellular proteins had comparable scores (Beta-2-microglobulin and FK506 binding protein 1A, with emPAI scores of 1.79 and 1.39, respectively). ORF12 encodes a soluble TNF receptor superfamily homologue which, like ORF134, was expected to be secreted from infected cells. Three unique peptides covering 16% of the ORF134 sequence were sequenced (Figure 3a). These peptides were distributed throughout ORF134 sequence (Figure 3b). The divergence existing between CyHV-3 IL-10 and carp IL-10 excludes the hypothesis that the peptides detected could be derived from carp IL-10 rather than from CyHV-3 ORF134. In addition to CyHV-3 ORF12 and ORF134, three additional viral proteins (ORF52, ORF116 and ORF119) were detected in the CyHV-3 secretome. All three proteins are potential membrane proteins (Table 2). The presence of these putative membrane proteins in the CyHV-3 secretome cannot be explained by remaining viral particles in the prepared concentrated extracellular medium, as none of these proteins is structural [31]. It is also unlikely that the presence of these proteins reflects cell lysis resulting from the viral infection. Indeed, in such a case, a higher number of viral proteins would be expected, in particular the most abundant ones [31]. Several viral proteins are expressed as two different forms, a membrane-anchored form and a secreted form, the latter generated by proteolytic cleavage of the former [42,43]. Further experiments are required to determine whether this phenomenon applies to the putative CyHV-3 membrane proteins detected in the secretome.

**Table 2:** CyHV-3 and host proteins identified by 2D-LC MS/MS in the supernatant of CyHV-3 infected CCB cells.

**Database**	**Accession number**	**Description**	**Predicted MW (kDa)**	**Mascot score**	**No. of matching spectra**	**emPAI**
		**CyHV-3 proteins**				
***Cyprinivirus***	gi|129560530	ORF12, TNF receptor superfamily homologue	19.2	671	14	1.49
	gi|129560652	ORF134, Interleukin 10 homologue	14.8	304	6	1.02
	gi|84181525	ORF116, predicted membrane glycoprotein	30.4	185	4	0.26
	gi|84181523	ORF119, putative uncharacterized protein containing an hydrophobic region	15.5	94	2	0.25
	gi|129560569	ORF52, predicted membrane glycoprotein	39.2	44	1	0.10
		**Host proteins (species origin)**				
***Bony vertebrates***	gi|122891218	Novel protein (zgc:103659) (*Danio rerio*)	51.7	410	13	0.42
	gi|136429	Trypsin (*Sus scrofa*)	25.1	281	9	0.53
	gi|297262447	Predicted keratin, type II cytoskeletal 1-like isoform 6 (*Macaca mulatta*)	65.3	268	3	0.12
	gi|37590349	Enolase 1, alpha (*D. rerio*)	47.4	255	8	0.35
	gi|326670662	Predicted collagen alpha-3(VI) chain-like (*D. rerio*)	11.3	227	8	0.17
	gi|51949771	Fibronectin 1b (*D. rerio*)	279.3	213	8	0.08
	gi|52218922	Pigment epithelium-derived factor precursor (*D. rerio*)	45.0	158	3	0.17
	gi|416696	Beta-2-microglobulin (*Cyprinus carpio*)	13.5	152	8	1.79
	gi|1351907	Serum albumin (*Bos taurus*)	71.2	149	11	0.36
	gi|395744345	Predicted keratin, type II cytoskeletal 1 (*Pongo abelii*)	25.8	139	3	0.15
	gi|229552	Albumin (*B. taurus*)	68.1	136	10	0.38
	gi|63102189	Pgd protein (D. rerio)	53.7	134	4	0.14
	gi|15718387	Gelatinase (*Paralichthys olivaceus*)	75.5	125	5	0.15
	gi|1703244	Fructose-bisphosphate aldolase C (*Carassius auratus*)	39.8	124	4	0.20
	gi|169154447	Fibronectin 1 (*D. rerio*)	275.6	117	5	0.05
	gi|15149946	Procollagen type I alpha 1 chain (*D. rerio*)	49.4	117	5	0.34
	gi|148726027	Cadherin 11, osteoblast (*D. rerio*)	88.9	112	5	0.13
	gi|4885063	Fructose-bisphosphate aldolase C (*Homo sapiens*)	39.8	107	2	0.20
	gi|28336	Mutant beta-actin (beta'-actin) (*H. sapiens*)	42.1	105	2	0.09
	gi|28317	Unnamed protein product (*H. sapiens*)	59.7	104	3	0.13
	gi|337758	Pre-serum amyloid P component (*H. sapiens*)	25.5	100	3	0.32
	gi|223582	Histone H4 (*H. sapiens*)	11.2	99	5	0.84
	gi|47971186	Carp C1q-like molecule (*C. carpio*)	20.3	98	2	0.19
	gi|223061	Ubiquitin (*Salmo sp.*)	8.5	92	4	0.31
	gi|27806751	Alpha-2-HS-glycoprotein precursor (*B. taurus*)	39.2	90	4	0.32
	gi|47086029	Myristoylated alanine-rich C kinase substrate 2 (*D. rerio*)	21.0	86	2	0.18
	gi|2133885	N-cadherin precursor (*D. rerio*)	87.4	80	3	0.09
	gi|18859555	Wnt inhibitory factor 1 precursor (*D. rerio*)	43.2	79	3	0.09
	gi|34595971	Prion-like protein 1 (*C. carpio*)	55.6	75	2	0.14
	gi|208609649	Collagen type I alpha 3 (*C. auratus*)	137.7	75	1	0.03
	gi|47085905	14-3-3 protein beta/alpha-B (*D. rerio*)	27.5	75	3	0.47
	gi|6644111	Nucleoside diphosphate kinase-Z1 (*D. rerio*)	17.4	72	2	0.22
	gi|16974825	Chain A, Solution Structure Of Calcium-Calmodulin N-Terminal Domain (*H. sapiens*)	8.5	70	2	0.49
	gi|41152406	FK506 binding protein 1A, 12kDa (*D. rerio*)	11.8	69	3	1.39
	gi|189527793	Predicted neuroblast differentiation-associated protein AHNAK (*D. rerio*)	642.1	65	2	0.01
	gi|37492	Alpha-tubulin (H. sapiens)	50.8	65	2	0.07
	gi|33989505	Tissue inhibitor of metalloproteinase 2b (*D. rerio*)	24.7	64	2	0.15
	gi|8176557	Heart fatty acid binding protein (*Anguilla japonica*)	15.3	61	1	0.26
	gi|37181	Tissue inhibitor of metalloproteinases, Type-2 (*H. sapiens*)	21.4	59	2	0.18
	gi|437972	Fibrillin-2 (*H. sapiens*)	334.8	59	1	0.01
	gi|37367051	Osteopontin (*D. rerio*)	23.2	53	1	0.16
	gi|45544646	Cold inducible RNA binding protein isoform 2 (*D. rerio*)	19.2	52	2	0.20
	gi|51328294	Fstl1b protein (*D. rerio*)	39.6	50	1	0.09
	gi|82245450	Triosephosphate isomerase B (*D. rerio*)	27.1	50	1	0.14
	gi|34014734	Clusterin (*D. rerio*)	52.5	50	1	0.07
	gi|47228578	Unnamed protein product (*Tetraodon nigroviridis*)	77.5	49	1	0.05

The MS data presented above demonstrate that CyHV-3 ORF134 encodes a protein that is abundantly secreted in the extracellular medium by infected cells. This observation is consistent with the hypothesis that ORF134 may be a functional IL-10 homologue playing a role in CyHV-3 pathogenesis [26].

### Production and characterization of CyHV-3 ORF134 recombinant strains

**Figure 4:**
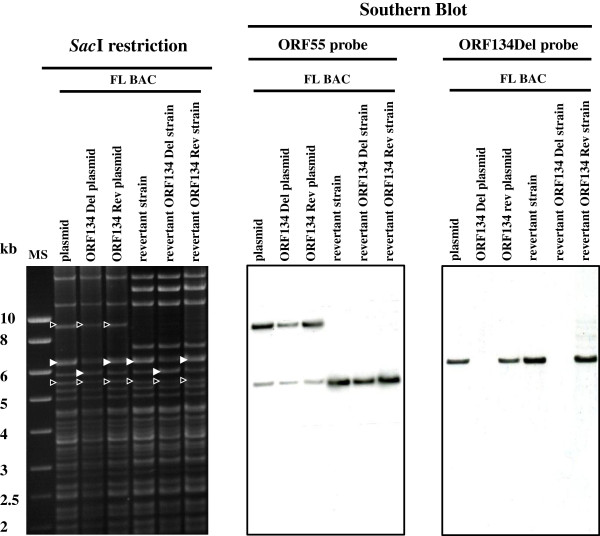
**Structural analysis of the FL BAC ORF134 recombinant plasmids and derived CyHV-3 recombinant strains.** The CyHV-3 FL BAC, FL BAC ORF134 Del and FL BAC ORF134 Rev plasmids and the genome of the FL BAC revertant, FL BAC revertant ORF134 Del and FL BAC revertant ORF134 Rev strains were analyzed by *Sac*I restriction (left panel) and further tested by southern blotting using probes corresponding to ORF55 (central panel) and ORF134 (right panel). White-outlined black arrowheads and white arrows indicate restriction fragments containing ORF55 and ORF134 loci, respectively. Marker sizes (MS) are indicated on the left.

**Figure 5:**
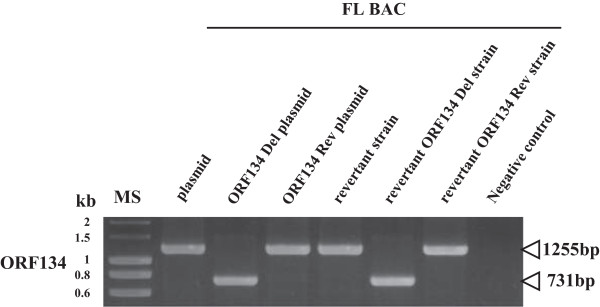
**PCR analysis of the FL BAC ORF134 recombinant plasmids and derived CyHV-3 recombinant strains.** The CyHV-3 FL BAC, FL BAC ORF134 Del and FL BAC ORF134 Rev plasmids and the genome of the FL BAC revertant, FL BAC revertant ORF134 Del and FL BAC revertant ORF134 Rev strains were analyzed by PCR using the forward primer ORF134outseqF and the reverse primer ORF134outseqR (Table 1). MS are indicated on the left.

**Figure 6:**
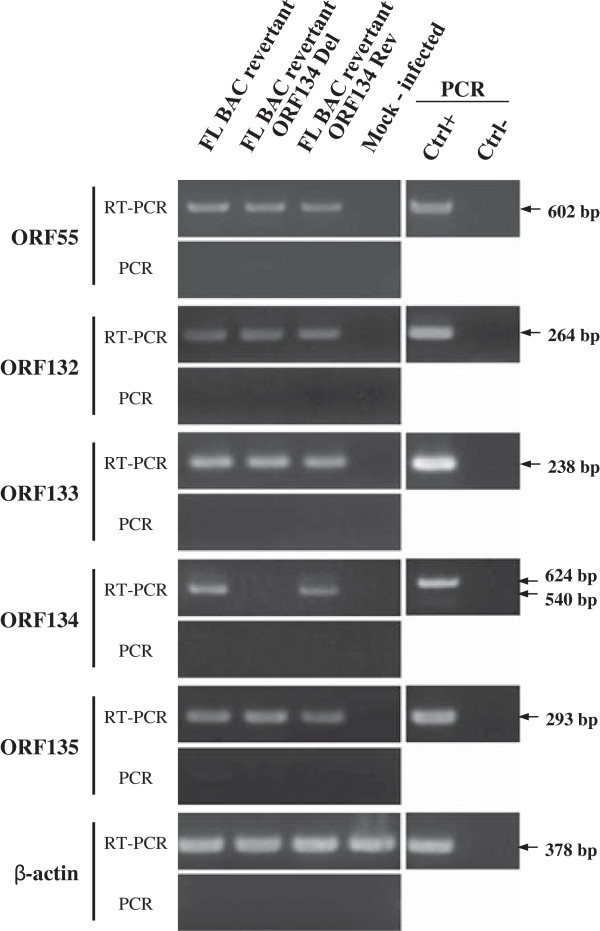
**Transcriptional analysis of CyHV-3 ORF134 genome region.** CCB cells were infected with the indicated recombinant strains at a MOI of 0.5 PFU/cell. Twenty-four hours post-infection, expression of CyHV-3 ORF55, ORF132, ORF133, ORF134, ORF135 and carp β-actin was studied by the RT-PCR approach described in the Materials and methods. On the left RT-PCR or PCR represent PCR products generated when the RT was performed or omitted from the reactions, respectively. On the right, control PCR reactions were performed using genomic DNA as template (Ctrl+) or no template (Ctrl-).

In order to investigate subsequently the importance of ORF134 in virus replication in vitro and pathogenesis in vivo, a CyHV-3 strain deleted for ORF134 (FL BAC revertant ORF134 Del strain) and a revertant strain (FL BAC revertant ORF134 Rev strain) were produced using BAC cloning and prokaryotic recombination technologies as described in the Materials and methods (Figure 1). The FL BAC plasmid was used as parental plasmid. A wild type strain (FL BAC revertant strain) was also reconstituted from the FL BAC plasmid. The molecular structures of the recombinant strains produced were confirmed by a combined *Sac*I restriction endonuclease and Southern blot approach targeting both ORF55 (the BAC cassette is inserted into the ORF55 locus) and ORF134 loci (Figure 4). In the three reconstituted strains, the ORF55 probe led to a single band corresponding to a 5.2 kb restriction fragment, demonstrating the reversion of ORF55 to wild type sequence (removal of the BAC cassette) [27]. In the FL BAC revertant and the FL BAC revertant ORF134 Rev, the ORF134Del probe led to a single band corresponding to a 6.3 kb restriction fragment consistent with the sequence of this region (6333kb). The absence of signal for the FL BAC revertant ORF134 Del demonstrated the deletion of ORF134. The molecular structure of the recombinants and the absence of contamination between strains was also controlled by PCR (Figure 5) and sequencing of the regions used to target recombination (data not shown). All approaches confirmed that the resulting recombinants have the correct molecular structure. Finally, using a RT-PCR approach, we controlled the process so that the deletion did not markedly affect the transcription of the ORFs located upstream and downstream of ORF134: ORF132, ORF133 and ORF135 (Figures 1a and 6). In these experiments, transcription of ORF55 was used as reference. For the three recombinants tested, transcripts of 602 bp, 264 bp, 238 bp and 293 bp were observed in infected cells for ORF55, ORF132, ORF133 and ORF135, respectively. No transcript was detected in mock-infected cells. When RT was omitted from the reactions, the product seen in infected cells was not detected, indicating that this product did not result from amplification of contaminant viral DNA. The three strains tested led to comparable signals for the four ORFs. Transcription analysis of ORF134 revealed that the FL BAC revertant and the FL BAC revertant ORF134 Rev expressed this ORF comparably, while no signal was detected for the FL BAC revertant ORF134 Del. Together, the results presented above demonstrate that the recombinants produced have the correct molecular structure and that the deletion of ORF134 has no marked polar effect on neighbor genes.

### Effect of ORF134 deletion on viral growth in vitro

**Figure 7:**
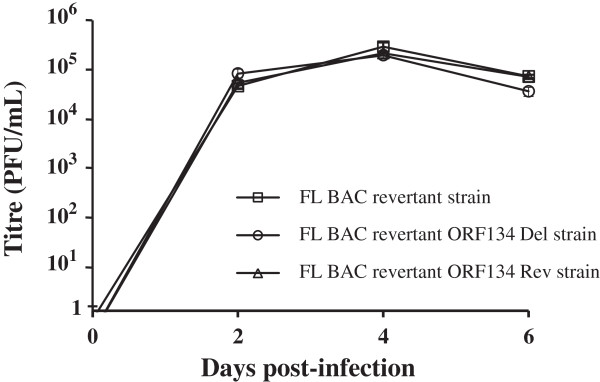
**Effect of ORF134 deletion on viral growth in vitro.** Replication kinetics of CyHV-3 ORF134 recombinant strains were compared with those of the FL BAC revertant strain using a multi-step growth assay (see Materials and methods). The data presented are the means ± standard errors of triplicate measurements.

In order to investigate the putative effects of the recombination processes on viral growth in vitro, the FL BAC revertant, the FL BAC revertant ORF134 Del and the FL BAC revertant ORF134 Rev were compared using a multi-step growth assay (Figure 7). All viruses tested exhibited similar growth curves (*P* ≤ 0.05), leading to the conclusion that ORF134 deletion does not affect viral growth in vitro (Figure 7). This observation is consistent with what has been reported for other vIL-10s [23,25]. Taken together, these results indicate that ORF134 is not essential for CyHV-3 replication in vitro and suggest that ORF134 exerts its biological functions in vivo.

### Effect of ORF134 deletion on CyHV-3 pathogenesis

**Figure 8:**
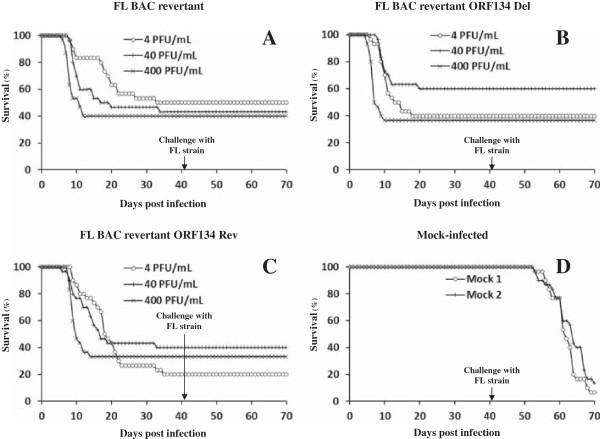
**Cumulative survival rates of common carp infected with CyHV-3 recombinant strains.** On day 0, common carp (*n* = 30 fish per tank), with an average weight of 3.8 g ± 1.5 g (mean ± SD), were mock-inoculated (2 tanks, panel **D**) or inoculated (panels **A**-**C**) by immersion for 2 h in water containing 4 PFU/mL, 40 PFU/mL or 400 PFU/mL of the indicated CyHV-3 strains. On day 42 post-infection (arrow), surviving fish were challenged by addition of two fish infected with the parental FL strain. Percentages of surviving carp are expressed according to days post-infection.

**Figure 9:**
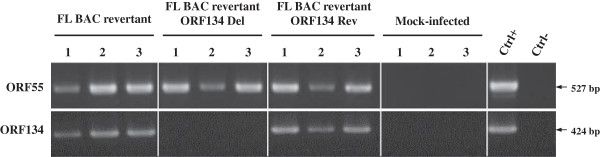
**PCR detection and characterization of CyHV-3 genomes recovered from infected carp.** The analyses reported in this figure are the follow-up of the experiment described in Figure 8. Three mock-infected carp (selected randomly before the challenge) and three dead carp from each of the groups infected with the FL BAC Revertant, FL BAC Revertant ORF134 Del and FL BAC Revertant ORF134 Rev strains were dissected. DNA was extracted from the kidney. PCRs were performed with the ORF55InF/ORF55stopR and ORF134InF/ORF134InR pairs of primers (Table 1). FL strain DNA and distilled water were used as positive (Ctrl+) and negative (Ctrl-) controls, respectively.

To investigate the importance of ORF134 in the pathogenesis of CyHV-3 disease, naïve common carp were inoculated by immersion in water containing the FL BAC revertant, FL BAC revertant ORF134 Del or FL BAC revertant ORF134 Rev strains (Figure 8). The three strains induced at comparable levels all the clinical signs associated with the disease, including apathy, folding of the dorsal fin, hyperemia, increased mucus secretions, skin lesions, suffocation, erratic swimming, and the loss of equilibrium. The mortality rate and the kinetics of mortality observed for the three strains were not significantly different. At necropsy, similar lesions were observed for the three strains including the discoloration of gill filaments, herpetic skin lesions, and necrotic nephritis. To control that the infection of all groups of fish was performed with the correct viral strain and to exclude any possibility of wild type virus spread among tanks, PCR assays were performed on three randomly selected dead fish from each infected group and three mock-infected fish randomly selected (Figure 9). The PCR results confirmed that each tank was infected with the correct strain and demonstrated the absence of viral spread between tanks. Next, to determine whether the ORF134 deletion affects the adaptive immune response developed by fish that survived primary infection; surviving fish were challenged by co-habitation with fish inoculated with the wild type FL strain (Figure 8). Independently of the viral strain used for the primary infection, none of the challenged fish developed CyHV-3 disease (Figure 8). In contrast, CyHV-3 disease developed in the two tanks that were initially mock-infected. Taken together, the results presented above suggest that ORF134 deletion does not affect CyHV-3 pathogenicity in common carp and the protective immune response developed by surviving fish.

**Figure 10:**
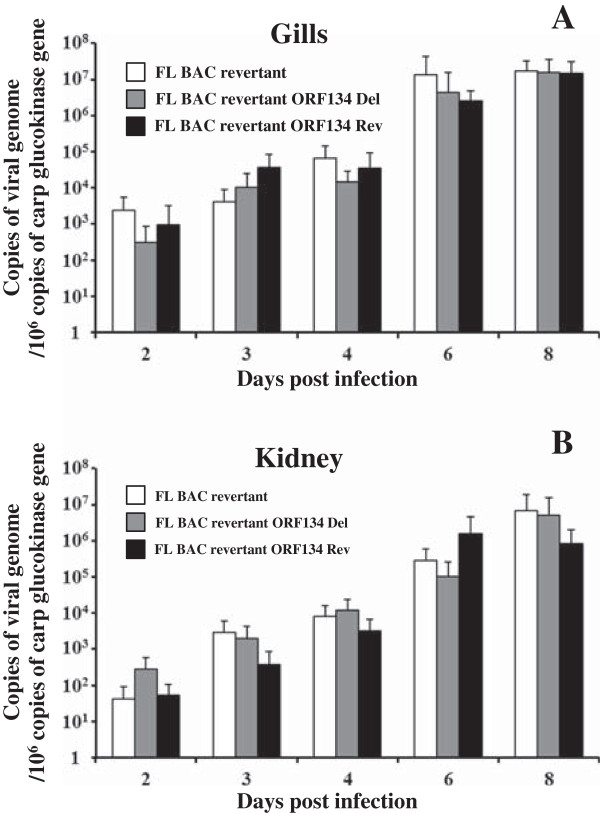
**Viral load in gill and kidney.** Common carp (*n* = 36 fish per tank), with an average weight of 10.7 g ± 3.2 g (mean ± SD), were inoculated by immersion for 2 h in water containing 100 PFU/mL of the indicated CyHV-3 strains. At different times post-inoculation, six infected fish were randomly selected per tank, euthanized and dissected. Six mock-infected fish were used as negative controls. Gill, kidney and spleen were harvested and stored in RNAlater® at −80 °C. DNA was extracted from gill (panel **A**) and kidney (panel **B**) and analyzed by real-time TaqMan PCR for quantification of viral genome copies. The results are expressed as the means ± SD of the data observed for the 6 fish analyzed per time point. Spleen were treated for quantification of carp gene expression by RT-qPCR (see Figure 11).

**Figure 11:**
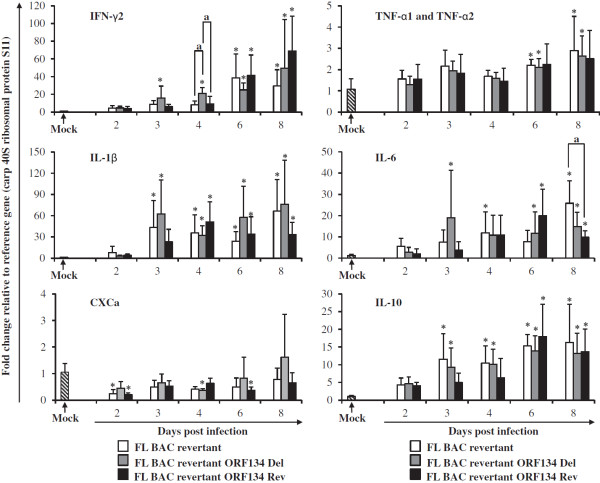
**RT-qPCR analysis of cytokines.** Kinetics of gene expression was measured in the spleen of mock-infected fish and fish infected with CyHV-3 ORF134 recombinants (see legend of Figure 10). Gene expression was normalized relative to the expression of the S11 protein of the 40S ribosomal subunit. Data are presented as mean ± SD (*n* = 6). Symbol (*) indicates statistical differences (*p* ≤ 0.05) observed between infected and mock-infected fish. Symbol (a) indicates statistical differences (*p* ≤ 0.05) observed for a specific time point between groups of fish infected with different CyHV-3 recombinants.

To further test these hypotheses, we investigated the effect of ORF134 deletion on viral load (Figure 10) and on cytokine expression (Figure 11) during CyHV-3 disease. Naïve common carp were inoculated by immersion in water containing the FL BAC revertant, FL BAC revertant ORF134 Del or FL BAC revertant ORF134 Rev strains (Figure 10). At different times after inoculation gill, kidney and spleen were collected from randomly selected fish. Viral loads were analyzed in gill and kidney by real-time TaqMan PCR (Figure 10) while cytokine expression was studied in spleen by RT-qPCR (Figure 11).

Real-time TaqMan PCR results demonstrated that fish infected with the three viral strains had statistically comparable viral loads in the gills and the kidney throughout the course of the experiment (Figure 10). Using the approach described in Figure 9, PCR reactions were performed on randomly selected fish demonstrated that each tank was infected with the correct strain and confirmed the absence of viral spread between tanks (data not shown). Together, these results suggested that ORF134 deletion has no effect on viral load during primary acute infection.

The spleen is one of the organs in which CyHV-3 is the most abundant during the course of acute infection [36]. It is also considered as one of the major lymphoid organ in teleost [44]. In order to study the effect of CyHV-3 ORF134 on the carp immune response, the kinetics of gene expression of the cytokines IFN-γ2, TNFα1, TNFα2, IL-1β, IL-6, CXCa and IL-10 were analyzed in spleen from fish infected with FL BAC revertant, FL BAC revertant ORF134 deleted and FL BAC revertant ORF134 Rev strains (Figure 11). Samples were collected over a period of 2 to 8 days post-infection and analyzed by RT-qPCR. The kinetics of expression of studied cytokines showed similar patterns to those observed previously [37]. Taking mock-infected fish as a reference, expression of several cytokines (IFN-γ2, IL-1β, IL-6, and IL-10) was up-regulated as early as day 3 post-infection. The most pronounced up-regulation was observed for IL-1β and IFN-γ2. We observed a moderate and late (day 6 and day 8 post-infection) up-regulation of TNFα1 and TNFα2. The expression level of CXCa in infected fish was comparable to mock-infected fish or even down-regulated for some strains at some time points. Importantly, the results presented in Figure 11 demonstrate that there is almost no difference in the expression levels of the cytokines studied between carp infected with three virus strains. The only significant differences observed between virus strains were for IFN-γ2 at day 4 post-inoculation and for IL-6 at day 8 post-inoculation. The expression level of IFN-γ2 at day 4 post-inoculation was significantly higher in fish infected with FL BAC revertant ORF134 deleted as compared to FL BAC revertant and FL BAC revertant ORF134 Rev strains. However, this difference was rather small and was not observed for the other sampling points, suggesting that it could reflect data variation rather than the expression of ORF134 biological activities. Supporting the latter hypothesis, the expression level of IL-6 at day 8 post-inoculation was significantly higher in the FL BAC revertant group as compared to the FL BAC revertant ORF134 Rev group. The absence of cross-contamination between tanks was controlled using the approach described in Figure 9 (data not shown). Together, these results suggested that ORF134 does not significantly affect the carp immune response under the experimental conditions used.

**Figure 12:**
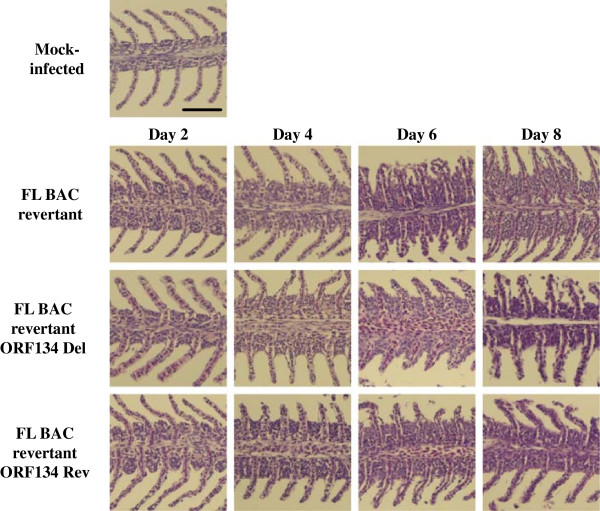
**Histopathological characterization of the lesions induced by CyHV-3 ORF134 recombinants in the gills.** Common carp (*n* = 20 fish per tank), with an average weight of 6 g ± 1.6 g (mean ± SD), were inoculated by immersion for 2 h in water containing 40 PFU/mL of the FL BAC revertant, FL BAC revertant ORF134 Del or FL BAC revertant ORF134 Rev strains. At different times post-inoculation (2, 4, 6 and 8 days), three infected fish were randomly selected per tank, euthanized and dissected. Three mock-infected fish were used as negative controls. Gills (present figure) and kidney (see Figure 13) were collected and processed for histological examination. Slides corresponding to each sampling point were grouped according to the viral strain used for the inoculation and were submitted to histopathological examinations by two independent examiners using a double-blind test mode. The images in this figure are representative of the analysis of one selected fish per group. Bar, 30 μm.

**Figure 13:**
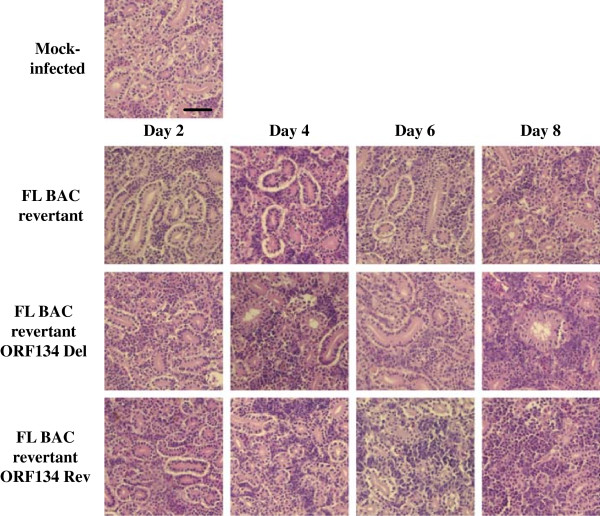
**Histopathological characterization of the lesions induced by CyHV-3 ORF134 recombinants in the kidney.** The fish infection methods are described in the legend of Figure 12.

Finally, to investigate further the effect of ORF134 in CyHV-3 pathogenesis, the lesions induced by the FL BAC revertant, FL BAC revertant ORF134 Del and FL BAC revertant ORF134 Rev strains were compared in the gills and the kidney at various time points after infection (Figures 12 and 13). Histopathological preparations were grouped according to the virus genotype used for the infection and the time point of sampling. The groups of slides were observed by two independent examiners using a double-blind test. The principal histopathological changes were observed in gill filaments. Gills from mock-infected fish exhibited a normal structure. However, a weak lymphocytic hyperplasia was observed for the three mock-infected fish at the basis of the secondary lamellae, leading to their fusion. Few eosinophilic granulocytes were also observed along the primary lamella. As early as 2 days post-infection, both examiners were able to discriminate the three groups of infected fish from the mock-infected group. For all three infected groups, we observed congestion of the secondary lamellae, infiltration of lymphocytes and histiocytes at the basis of secondary lamellae further increasing their fusion. With the exception of one fish from the FL BAC revertant ORF134 Del group that exhibited weaker histopathological changes (see Figure 12, Day 2), the two other fish from this group expressed changes comparable to those observed in the two other infected groups. The absence of differences between the three viral groups was confirmed at the latter time points. At day 4 post-infection, all fish expressed comparable increased lymphocytic and histocytic infiltrate at the basis of the secondary lamellae. In some fish, an increase of eosinophilic granulocytes was observed (FL BAC revertant: 2 out of 3 fish; FL BAC revertant ORF134 Del: 2 out of 3 fish and FL BAC revertant ORF134 Rev strains 1 out of 3 fish). In comparison to day 2 post-infection, the infiltrate was more pronounced and the congestion was associated with edema of the secondary lamellae. The intensity of the lesions increased comparably in all three groups at latter time-points (Day 6 and Day 8). The infiltrate mainly lymphocytic induced the fusion of the lamellae on approximately 2/3 of their length. The respiratory epithelium exhibited hyperplasia and necrosis, associated in few cells with intranuclear inclusion bodies. Compared to day 6 post-infection, the infiltrate observed on day 8 was slightly reduced while the edema and the necrosis were increased. The lesions induced by the three recombinant strains were also compared in the kidney (Figure 13). The lesions observed in this organ were less obvious than in the gills. On day 2 post-infection, infected groups could not be differentiated from the mock-infected one. The diversity and the abundance of hematopoietic cells were normal. However, a slight increase of eosinophilic cells was observed in nearly all groups. Vacuolization of the epithelium was observed in all preparations, and was considered to be a preparation artifact. Starting on day 4 post-infection, both examiners were able to discriminate the three infected groups from the mock-infected one. However, they could not differentiate the three infected groups. Comparable proliferation of the hematopoietic cells, mainly lymphocytic and eosinophilic, was observed in all infected groups. The proliferation increased further on day 6 and 8. Intranuclear inclusion bodies were observed in a few hematopoietic cells on days 6 and 8, and in few epithelial cells on day 8. The absence of cross-contamination between tanks was controlled using the approach described in Figure 9 (data not shown).

## Discussion

The present study was devoted to CyHV-3 ORF134, which encodes a potential vIL-10. We confirmed that ORF134 is transcribed as a spliced E-L gene (Figure 2). We also demonstrated for the first time that it is one of the most abundant proteins of the CyHV-3 secretome (Table 1) and that ORF134 is essential neither for viral replication in vitro nor for virulence in vivo. The latter conclusion relied on the observations that an ORF134 deleted strain could not be differentiated from its parental and revertant strains based on induced clinical signs and mortality rate (Figure 8), kinetic of viral load in gills and kidney (Figure 10), kinetic of cytokine expression in the spleen (Figure 11) and histological examination of gill and kidney (Figure 12).

As described in the introduction, cellular IL-10 is a pleiotropic immunomodulatory cytokine with both immunostimulatory and immunosuppressive properties [14]. Virally encoded IL-10 homologues have been reported in several members of the *Poxviridae* family and the *Herpesvirales* order [19-21]. Numerous molecular and in vitro studies suggest that there has been adaptive evolution of viral IL-10 following capture through positive selection to retain properties most beneficial for the virus life cycle. However, very few studies have addressed the role of viral IL-10 in vivo by comparison of a wild type strain and derived deleted and revertant strains. This approach, which is the only one that can test the in vivo biological relevance of a gene, has been performed for only two viruses: rhesus cytomegalovirus (rhesus CMV) and Orf virus (ORFV) [23-25]. For both viruses, deletion of viral IL-10 induced virus attenuation and modulation of the host anti-viral innate immune response.

The results of the present study demonstrate that the IL-10 homologue encoded by CyHV-3 does not affect significantly its virulence in common carp (Figure 8) or the host innate immune response (Figure 11). However, a recent study based on an in vivo artificial model suggested that CyHV-3 ORF134 encodes a functional vIL-10 [26]. As IL-10 is known to induce a transient neutrophilia and monocytosis in addition to T cell suppression [45], these authors tested the in vivo functionality of CyHV-3 encoded IL-10 by injection of zebrafish embryos with mRNA encoding CyHV-3 ORF134 and analysis by whole-mount *in situ* hybridization (using a pan-leukocyte marker lysozyme at 56 hours post-fertilization before development of T cells). A slight but statistically significant increase in the number of lysozyme positive cells was observed in embryos injected with CyHV-3 ORF134 mRNA compared to control embryos. The effect observed was inhibited by down regulation of the IL-10 receptor long chain by a specific morpholino. These data suggested that CyHV-3 ORF134 encodes a functional vIL-10. Importantly, the ORF134 sequence used in this study is identical to the sequence encoded by the CyHV-3 strain used in the present study. Various hypotheses could explain the apparent paradox between the functional effect reported by Sunarto et al. and the lack of effect of deleting ORF134 described in the present study [26].

Firstly, it is possible that the slight effect observed by Sunarto et al. using optimal artificial conditions (overexpression of ORF134, no inflammatory stimulation by the viral infection, a rather immature host immune system) has no significant biological relevance during a real viral infection of carp. Secondly, it is possible that the role of ORF134 is strictly restricted to latency and viral reactivation. This hypothesis is inconsistent with the higher level of ORF134 expression observed during acute infection compared to those observed during latency and reactivation [26]. However, experiments are in progress to determine whether ORF134 deletion affects viral load during latency and/or the ability of the virus to reactivate and to be excreted. Thirdly, it may be that ORF134 expression product has a biological activity in zebrafish but not in common carp. This hypothesis is related to the still unknown origin of CyHV-3. Indeed, the rapid emergence of CyHV-3 in the common and koi carp population during the late 90s and the relatively low polymorphism existing between CyHV-3 isolates suggest that CyHV-3 is the consequence from a recent host-jump from a yet unidentified fish species to common and koi carp. According to this evolutionary scenario, it could be that ORF134 is functional in the CyHV-3 original host species and closely related species but not in the recently colonized common and koi carp species.

In conclusion, the present study addressed for the first time the in vivo role of a vIL-10 encoded by a member of the family *Alloherpesviridae*. It demonstrates that CyHV-3 ORF134 does not contribute significantly to viral growth in vitro or to virulence in vivo under the conditions tested. However, it is possible that this protein is important under circumstances that were not recapitulated in the present laboratory setting.

## Abbreviations

AngHV-1: Anguilid herpesvirus 1; BAC: Bacterial artificial chromosome; CCB: *Cyprinus carpiocarpio* brain cell; CHX: Cycloheximide; CyHV-3: Cyprinid herpesvirus-3; Ct: Threshold cycle; DMEM: Dulbecco’s modified essential medium; E: Early; EBV: Epstein-Barr virus; EGFP: Enhanced green fluorescent protein; galK: Galactokinase; HCMV: Human cytomegalovirus; IE: Immediate early; IFN: Interferon; IL-10: Interleukin-10; KHV: Koi herpesvirus; L: Late; MOI: Multiplicity of infection; MS: Mass spectrometry; ORF: Open reading frame; ORFV: Orf virus; PBS: Phosphate buffered saline; PAA: Phosphonoacetic acid; PFU: Plaque forming unit; Rhesus CMV: Rhesus cytomegalovirus; RT-PCR: Reverse transcription PCR; RT-qPCR: Real-time quantitative PCR; TK: Thymidine kinase; vIL-10: Virally encoded IL-10 homologues.

## Competing interests

The authors declare that they have no competing interests.

## Authors’ contributions

PO did most of the experiments. PO, KR, MB, AR, MR, GF, BC and AV contributed to the design of the study. PO and KR drafted the figures. BL and RW performed proteomic analyses. SC produced recombination cassettes. Statistical analyses were performed by KR. AV conceived the study and drafted the manuscript. All authors read and approved the final manuscript.

## References

[B1] Bretzinger A Fischer-Scherl T Oumouna M Hoffmann R Truyen U Mass mortalities in Koi carp, Cyprinus carpio, associated with gill and skin disease Bull Eur Assoc Fish Pathol 1999 19 182 185

[B2] Hedrick R Gilad O Yun S Spangenberg J Marty G Nordhausen R Kebus M Bercovier H Eldar A A herpesvirus associated with mass mortality of juvenile and adult koi, a strain of common carp J Aquat Anim Health 2000 12 44 57 10.1577/1548-8667(2000)012<0044:AHAWMM>2.0.CO;2 28880775

[B3] Hedrick RP Movement of pathogens with the international trade of live fish: problems and solutions Rev Sci Tech 1996 15 523 531 8890378 10.20506/rst.15.2.938

[B4] Haenen O Way K Bergmann S Ariel E The emergence of koi herpesvirus and its significance to European aquaculture Bull Eur Assoc Fish Pathol 2004 24 293 307

[B5] Michel B Fournier G Lieffrig F Costes B Vanderplasschen A Cyprinid herpesvirus 3 Emerg Infect Dis 2010 16 1835 1843 10.3201/eid1612.100593 21122210 PMC3294573

[B6] Aoki T Hirono I Kurokawa K Fukuda H Nahary R Eldar A Davison AJ Waltzek TB Bercovier H Hedrick RP Genome sequences of three koi herpesvirus isolates representing the expanding distribution of an emerging disease threatening koi and common carp worldwide J Virol 2007 81 5058 5065 10.1128/JVI.00146-07 17329333 PMC1900211

[B7] Davison AJ Kurobe T Gatherer D Cunningham C Korf I Fukuda H Hedrick RP Waltzek TB Comparative genomics of carp herpesviruses J Virol 2013 87 2908 2922 10.1128/JVI.03206-12 23269803 PMC3571366

[B8] Waltzek TB Kelley GO Stone DM Way K Hanson L Fukuda H Hirono I Aoki T Davison AJ Hedrick RP Koi herpesvirus represents a third cyprinid herpesvirus (CyHV-3) in the family Herpesviridae J Gen Virol 2005 86 1659 1667 10.1099/vir.0.80982-0 15914843

[B9] Davison AJ Eberle R Ehlers B Hayward GS McGeoch DJ Minson AC Pellett PE Roizman B Studdert MJ Thiry E The order Herpesvirales Arch Virol 2009 154 171 177 10.1007/s00705-008-0278-4 19066710 PMC3552636

[B10] McGeoch DJ Rixon FJ Davison AJ Topics in herpesvirus genomics and evolution Virus Res 2006 117 90 104 10.1016/j.virusres.2006.01.002 16490275

[B11] Ilouze M Dishon A Kahan T Kotler M Cyprinid herpes virus-3 (CyHV-3) bears genes of genetically distant large DNA viruses FEBS Lett 2006 580 4473 4478 10.1016/j.febslet.2006.07.013 16860321

[B12] Savan R Igawa D Sakai M Cloning, characterization and expression analysis of interleukin-10 from the common carp, Cyprinus carpio L Eur J Biochem 2003 270 4647 4654 10.1046/j.1432-1033.2003.03854.x 14622251

[B13] Zhang DC Shao YQ Huang YQ Jiang SG Cloning, characterization and expression analysis of interleukin-10 from the zebrafish (Danio rerion) J Biochem Mol Biol 2005 38 571 576 10.5483/BMBRep.2005.38.5.571 16202237

[B14] Sabat R Grutz G Warszawska K Kirsch S Witte E Wolk K Geginat J Biology of interleukin-10 Cytokine Growth Factor Rev 2010 21 331 344 10.1016/j.cytogfr.2010.09.002 21115385

[B15] de Waal MR Abrams J Bennett B Figdor CG de Vries JE Interleukin 10(IL-10) inhibits cytokine synthesis by human monocytes: an autoregulatory role of IL-10 produced by monocytes J Exp Med 1991 174 1209 1220 10.1084/jem.174.5.1209 1940799 PMC2119001

[B16] Fiorentino DF Zlotnik A Mosmann TR Howard M O’Garra A IL-10 inhibits cytokine production by activated macrophages J Immunol 1991 147 3815 3822 1940369

[B17] Moore KW de Waal MR Coffman RL O’Garra A Interleukin-10 and the interleukin-10 receptor Annu Rev Immunol 2001 19 683 765 10.1146/annurev.immunol.19.1.683 11244051

[B18] Couper KN Blount DG Riley EM IL-10: the master regulator of immunity to infection J Immunol 2008 180 5771 5777 18424693 10.4049/jimmunol.180.9.5771

[B19] Slobedman B Barry PA Spencer JV Avdic S Abendroth A Virus-encoded homologs of cellular interleukin-10 and their control of host immune function J Virol 2009 83 9618 9629 10.1128/JVI.01098-09 19640997 PMC2747999

[B20] Kotenko SV Pestka S Oppenheim JJ, Feldmann M Viral IL-10 variants Cytokine reference: a compendium of cytokines and other mediators of host defence 2001 New York: Academic Press

[B21] Hughes AL Origin and evolution of viral interleukin-10 and other DNA virus genes with vertebrate homologues J Mol Evol 2002 54 90 101 10.1007/s00239-001-0021-1 11734902

[B22] van Beurden SJ Forlenza M Westphal AH Wiegertjes GF Haenen OL Engelsma MY The alloherpesviral counterparts of interleukin 10 in European eel and common carp Fish Shellfish Immunol 2011 31 1211 1217 10.1016/j.fsi.2011.08.004 21907290

[B23] Chang WL Barry PA Attenuation of innate immunity by cytomegalovirus IL-10 establishes a long-term deficit of adaptive antiviral immunity ProcNatlAcadSci USA 2010 107 22647 22652 10.1073/pnas.1013794108 PMC3012498 21149711

[B24] Eberhardt MK Chang WL Logsdon NJ Yue Y Walter MR Barry PA Host immune responses to a viral immune modulating protein: immunogenicity of viral interleukin-10 in rhesus cytomegalovirus-infected rhesus macaques PLoS One 2012 7 e37931 10.1371/journal.pone.0037931 22655082 PMC3360012

[B25] Fleming SB Anderson IE Thomson J Deane DL McInnes CJ McCaughan CA Mercer AA Haig DM Infection with recombinant orf viruses demonstrates that the viral interleukin-10 is a virulence factor J Gen Virol 2007 88 1922 1927 10.1099/vir.0.82833-0 17554023

[B26] Sunarto A Liongue C McColl KA Adams MM Bulach D Crane MSJ Schat KA Slobedman B Barnes AC Ward AC Walker PJ Koi herpesvirus encodes and expresses a functional interleukin-10 J Virol 2012 86 11512 11520 10.1128/JVI.00957-12 22896613 PMC3486318

[B27] Costes B Fournier G Michel B Delforge C Raj VS Dewals B Gillet L Drion P Body A Schynts F Lieffrig F Vanderplasschen A Cloning of the koi herpesvirus genome as an infectious bacterial artificial chromosome demonstrates that disruption of the thymidine kinase locus induces partial attenuation in Cyprinus carpio koi J Virol 2008 82 4955 4964 10.1128/JVI.00211-08 18337580 PMC2346741

[B28] Markine-Goriaynoff N Gillet L Karlsen OA Haarr L Minner F Pastoret P-P Fukuda M Vanderplasschen A The core 2 β-1, 6-N-acetylglucosaminyltransferase-M encoded by bovine herpesvirus 4 is not essential for virus replication despite contributing to post-translational modifications of structural proteins J Gen Virol 2004 85 355 367 10.1099/vir.0.19715-0 14769893

[B29] Ilouze M Dishon A Kotler M Coordinated and sequential transcription of the cyprinid herpesvirus-3 annotated genes Virus Res 2012 169 98 106 10.1016/j.virusres.2012.07.015 22841491

[B30] Ilouze M Dishon A Kotler M Down-regulation of the cyprinid herpesvirus-3 annotated genes in cultured cells maintained at restrictive high temperature Virus Res 2012 169 289 295 10.1016/j.virusres.2012.07.013 22841492

[B31] Michel B Leroy B Stalin Raj V Lieffrig F Mast J Wattiez R Vanderplasschen AF Costes B The genome of cyprinid herpesvirus 3 encodes 40 proteins incorporated in mature virions J Gen Virol 2010 91 452 462 10.1099/vir.0.015198-0 19846671

[B32] Mastroleo F Leroy B Van Houdt RS Heeren C Mergeay M Hendrickx L Wattiez R Shotgun proteome analysis of Rhodospirillum rubrum S1H: integrating data from gel-free and gel-based peptides fractionation methods J Proteome Res 2009 8 2530 2541 10.1021/pr900007d 19243122

[B33] Ishihama Y Oda Y Tabata T Sato T Nagasu T Rappsilber J Mann M Exponentially modified protein abundance index (emPAI) for estimation of absolute protein amount in proteomics by the number of sequenced peptides per protein Mol Cell Proteomics 2005 4 1265 1272 10.1074/mcp.M500061-MCP200 15958392

[B34] Warming S Costantino N Court DL Jenkins NA Copeland NG Simple and highly efficient BAC recombineering using galK selection Nucleic Acids Res 2005 33 e36 10.1093/nar/gni035 15731329 PMC549575

[B35] Costes B Raj VS Michel B Fournier G Thirion M Gillet L Mast J Lieffrig F Bremont M Vanderplasschen A The major portal of entry of koi herpesvirus in Cyprinus carpio is the skin J Virol 2009 83 2819 2830 10.1128/JVI.02305-08 19153228 PMC2655586

[B36] Gilad O Yun S Zagmutt-Vergara FJ Leutenegger CM Bercovier H Hedrick RP Concentrations of a Koi herpesvirus (KHV) in tissues of experimentally infected Cyprinus carpio koi as assessed by real-time TaqMan PCR Dis Aquat Organ 2004 60 179 187 15521316 10.3354/dao060179

[B37] Rakus KL Irnazarow I Adamek M Palmeira L Kawana Y Hirono I Kondo H Matras M Steinhagen D Flasz B Brogden G Vanderplasschen A Aoki T Gene expression analysis of common carp (Cyprinus carpio L.) lines during Cyprinid herpesvirus 3 infection yields insights into differential immune responses Dev Comp Immunol 2012 37 65 76 10.1016/j.dci.2011.12.006 22212509

[B38] Livak KJ Schmittgen TD Analysis of relative gene expression data using real-time quantitative PCR and the 2^− ΔΔCT^ Method Methods 2001 25 402 408 10.1006/meth.2001.1262 11846609

[B39] Dewals B Boudry C Gillet L Markine-Goriaynoff N De Leval L Haig D Vanderplasschen A Cloning of the genome of Alcelaphine herpesvirus 1 as an infectious and pathogenic bacterial artificial chromosome J Gen Virol 2006 87 509 517 10.1099/vir.0.81465-0 16476972

[B40] Fleming SB McCaughan CA Andrews AE Nash AD Mercer AA A homolog of interleukin-10 is encoded by the poxvirus orf virus J Virol 1997 71 4857 4861 9151886 10.1128/jvi.71.6.4857-4861.1997 PMC191714

[B41] Lockridge KM Zhou SS Kravitz RH Johnson JL Sawai ET Blewett EL Barry PA Primate cytomegaloviruses encode and express an IL-10-like protein Virology 2000 268 272 280 10.1006/viro.2000.0195 10704336

[B42] Drummer HE Studdert MJ Crabb BS Equine herpesvirus-4 glycoprotein G is secreted as a disulphide-linked homodimer and is present as two homodimeric species in the virion J Gen Virol 1998 79 1205 1213 9603336 10.1099/0022-1317-79-5-1205

[B43] Costes B Ruiz-Arguello MB Bryant NA Alcami A Vanderplasschen A Both soluble and membrane-anchored forms of Felid herpesvirus 1 glycoprotein G function as a broad-spectrum chemokine-binding protein J Gen Virol 2005 86 3209 3214 10.1099/vir.0.81388-0 16298965

[B44] Rombout JH Huttenhuis HB Picchietti S Scapigliati G Phylogeny and ontogeny of fish leucocytes Fish Shellfish Immunol 2005 19 441 455 10.1016/j.fsi.2005.03.007 15890532

[B45] Chernoff AE Granowitz EV Shapiro L Vannier E Lonnemann G Angel JB Kennedy JS Rabson AR Wolff SM Dinarello CA A randomized, controlled trial of IL-10 in humans. Inhibition of inflammatory cytokine production and immune responses J Immunol 1995 154 5492 5499 7730651

